# Impaired cerebrovascular autoregulation in patients with severe sepsis and sepsis-associated delirium

**DOI:** 10.1186/cc11665

**Published:** 2012-10-04

**Authors:** Patrick Schramm, Klaus Ulrich Klein, Lena Falkenberg, Manfred Berres, Dorothea Closhen, Konrad J Werhahn, Matthias David, Christian Werner, Kristin Engelhard

**Affiliations:** 1 Department of Anaesthesiology, University Medical Centre of the Johannes Gutenberg-University Mainz, Langenbeckstraße 1, 55131 Mainz, Germany; 2 Institute of Medical Biometry, Epidemiology and Informatics (IMBEI), University Medical Centre of the Johannes Gutenberg-University Mainz, Langenbeckstraße 1, 55131 Mainz, Germany and RheinAhrCampus Remagen, Germany; 3 Department of Neurology, University Medical Centre of the Johannes Gutenberg-University Mainz, Langenbeckstraße 1, 55131 Mainz, Germany; 4 Department of Anaesthesiology, Intensive Care Medicine and Pain Therapy, Vienna General Hospital, University Vienna, Währinger Gürtel 18-20, 1090 Wien, Austria

## Abstract

**Introduction:**

Sepsis-associated delirium (SAD) increases morbidity in septic patients and, therefore, factors contributing to SAD should be further characterized. One possible mechanism might be the impairment of cerebrovascular autoregulation (AR) by sepsis, leading to cerebral hypo- or hyperperfusion in these haemodynamically unstable patients. Therefore, the present study investigates the relationship between the incidence of SAD and the status of AR during sepsis.

**Methods:**

Cerebral blood flow velocity was measured using transcranial Doppler sonography and was correlated with the invasive arterial blood pressure curve to calculate the index of AR Mx (Mx>0.3 indicates impaired AR). Mx was measured daily during the first 4 days of sepsis. Diagnosis of a SAD was performed using the confusion assessment method for ICU (CAM-ICU) and, furthermore the predominant brain electrical activity in electroencephalogram (EEG) both at day 4 after reduction of sedation to RASS >-2.

**Results:**

30 critically ill adult patients with severe sepsis or septic shock (APACHE II 32 ± 6) were included. AR was impaired at day 1 in 60%, day 2 in 59%, day 3 in 41% and day 4 in 46% of patients; SAD detected by CAM-ICU was present in 76 % of patients. Impaired AR at day 1 was associated with the incidence of SAD at day 4 (p = 0.035).

**Conclusions:**

AR is impaired in the great majority of patients with severe sepsis during the first two days. Impaired AR is associated with SAD, suggesting that dysfunction of AR is one of the trigger mechanisms contributing to the development of SAD.

**Trial registration:**

clinicalTrials.gov ID NCT01029080

## Introduction

Intensive care unit (ICU) patients with sepsis commonly develop neurological deficits as part of multi organ failure. A frequent form of central nervous system dysfunction is sepsis-associated delirium (SAD) that can be observed in septic patients [1]. According to the American Psychiatric Association's Diagnostic and Statistical Manual, 4th edition (DSM-IV), delirium can be defined as a rapidly evolving medical condition caused by a combination of disturbance of consciousness and change in cognition or confusion that is not accounted for by pre-existing dementia. Most importantly, SAD could be associated with increased morbidity [2].

Beside the inflammatory pathway many other mechanisms have been discussed that might cause SAD, such as microvascular dysfunction [3-6]. Laboratory and clinical investigations have revealed that cerebral perfusion is reduced during sepsis and cerebrovascular autoregulation (AR) may be impaired [7,8]. All ICU patients who die from septic shock have been shown to have ischemic brain lesions and furthermore, cerebral haemorrhage has been found in 26% of patients on neuropathologic examination after the patients died [9]. To maintain adequate brain function and neuronal integrity, cerebral blood flow (CBF) under physiologic circumstances is autoregulated within a wide range of cerebral perfusion pressure. When AR is disturbed, low cerebral perfusion pressures can lead to global cerebral ischaemia, while high cerebral perfusion pressures can cause haemorrhage and brain oedema. Both conditions may lead to neuronal damage that can potentially trigger SAD. In a small investigation of 16 patients impaired AR 48 hours after the onset of sepsis was found to be associated with SAD [10]. However, this study did not analyse the early time course after the onset of sepsis, measured AR once, and included only a few patients. Therefore, in the present study investigation of the status of AR began within 24 hours after the first signs of sepsis became obvious in 30 patients, the measurements were continued for four days, and the results tested for correlation with the occurrence of SAD.

## Materials and methods

Following ethical care committee approval of Rhineland-Palatinate (approval number: 837.435.08) and written informed consent from legal surrogates, 30 adult patients were included in the study. All patients had clinical diagnoses of severe sepsis or septic shock as defined by the international sepsis definition conference [11]. Exclusion criteria were pre-existing neurological or psychiatric diseases, intracranial infection, traumatic brain injury and pregnancy. Patient recruitment and first measurements were done within the first 24 hours after the diagnosis of sepsis was made.

Patient management was performed according to the international guidelines for management of severe sepsis and septic shock [12]. In all patients, the Acute Physiology and Chronic Health Evaluation II (APACHE II) score was evaluated at day 1 and the Sequential Organ Failure Assessment (SOFA) - score was evaluated daily. All patients included were sedated and ventilated at the ICU. Sedation was performed using propofol and sufentanil to achieve a target Richmond Agitation and Sedation Scale (RASS) score of -3 to 0. Mandatory ventilation with a tidal volume of 6 ml/Kg predicted body weight was performed. Ventilator frequency was adapted to maintain arterial partial pressure of carbon dioxide (paCO_2_) between 4.7 and 6.0 kPa (35 and 50 mmHg). Inspiratory oxygen fraction and positive end expiratory pressure were individually adjusted to achieve an arterial partial pressure of oxygen (paO_2_) > 9.0 kPa (70 mmHg). After stabilisation of the cardiopulmonary condition sedation was reduced and ventilation was switched to an assisted spontaneous breathing mode aiming for the same ventilatory settings. Invasive arterial blood pressure and the haemodynamic monitoring were measured by pulse contour analysis using a PiCCO^®^-Catheter (Pulsion Medical Systems AG, Munich, Germany). According to the local sepsis treatment protocol, saline solution was used to reach a global end-diastolic volume index of 650 to 800 ml/m^2^, norepinephrine was used to reach a mean arterial blood pressure > 65 mmHg, dobutamine was used to reach a target cardiac index > 3.5 l/min/m^2 ^and a central venous oxygen saturation > 70 %. Serum lactate level was measured to monitor adequate sepsis treatment.

### Transcranial Doppler ultrasound and cerebrovascular autoregulation

Measurement of the dynamic AR was performed daily for the first four days using transcranial Doppler (TCD) ultrasound. Cerebral blood flow velocities (CBFV) in both middle cerebral arteries were measured using 2-MHz TCD-probes (Doppler Box^®^, DWL, Sipplingen, Germany). The probes were positioned over the temporal bone window above the zygomatic arch and were fixed with a special frame (DiaMon^®^, DWL). This procedure ensured that the angle and the individual depth of insonation were constant during the daily investigation.

The index of AR was calculated using the software ICM+ (Cambridge Enterprise, University of Cambridge, UK). Spontaneous variations in CBFV and arterial blood pressure were measured over a 60-minute period, digitized and then processed calculating the time-averaged values using waveform time integration for 6-s intervals. The index of cerebrovascular autoregulation (Mx) was calculated as a Pearson's correlation coefficient between 30 samples of mean CBFV and mean arterial pressure (MAP) [13]. Positive association between variations in blood pressure and CBFV (positive values of Mx) indicates passive dependence of cerebral blood flow, and therefore impaired AR. Negative or zero values of Mx imply active cerebrovascular responses to changes in blood pressure and therefore preserved AR. The accepted cutoff level to discriminate between intact and impaired AR was 0.3 and values above 0.3 indicated impaired AR [14]. Mx was calculated for both brain hemispheres and the mean of both values over the time of measurement was used for the analysis. This method needs no further manipulation, such as changes of vasopressor dosing or thigh cuff deflating, which potentially lead to further complications.

Delirium as a clinical diagnosis was assessed using the confusion assessment method for the ICU (CAM-ICU) [2,4]. The CAM-ICU is a validated examination score to diagnose delirium in mechanically ventilated patients [15]. The CAM-ICU was assessed at day 4 during measurement of AR when sedative medication was temporarily reduced, to reach a RASS score greater than -2. In addition, electrophysiological evidence compatible with the diagnosis of delirium was gathered using electroencephalography (EEG) (Trex, XLTEK™, Oakville, Ontario, Canada). The EEG was recorded at day 4 after reduction of sedation (RASS score -2 to 0) with 16 surface electrodes placed according to the international 10/20-system for standardised placement and impedance level below 5 kΩ. EEGs were evaluated and interpreted by an EEG board-certified neurologist. All EEGs were classified by severity based on the predominant waveform from grade I to IV with I° = theta-delta-rhythm, II° = delta rhythm, III° = triphasic waveforms, and IV° = burst-suppression (modified as previously reported [16]).

### Laboratory analysis

For assessment of the severity of inflammation, serum concentrations of C-reactive protein (CRP) and procalcitonin (PCT) were measured daily. Furthermore, serum concentrations of neuron-specific enolase (NSE) and S100 were determined daily.

### Statistical analysis

Physiological data were expressed as the mean ± SD or the median (minimum, maximum) for nonparametric data. The primary endpoint of the present study was to investigate the association between the status of AR at day 1 and SAD at day 4. A power analysis calculated a need for 20 patients, which was increased to 30 because of the expected inhomogeneous distribution of the incidence of SAD. Data on AR and SAD were dichotomized in intact/impaired AR and SAD/non-SAD and then analysed using the Fisher exact test for the primary target (association between AR and SAD), and the Kendall-Tau-c test to compare the diagnosis of SAD using either CAM-ICU or EEG. To investigate whether other factors correlate with SAD, analysis of variance (ANOVA) correlations between AR, APACHE II scores, age and serum markers were performed and furthermore, spearman-Rho-correlations between Mx and serum markers and paCO_2 _by day (SPSS, version 19, SPSS Inc., Chicago, Illinois, USA). A *P*-value < 0.05 was considered statistically significant.

## Results

Thirty patients, five with severe sepsis and twenty-five with septic shock, APACHE II score of 32 ± 6, with a mean age of 64 ± 17 years were included in the study (Table 1). All patients required cardiovascular support and were sedated throughout the investigation using propofol and sufentanil. One patient died at day 2. Physiological data are represented in Table 2.

**Table 1:** Demographic characteristics

Patient number	Septic focus	Hospital stay	APACHE II score	Mx day1	Mx day 2	Mx day 3	Mx day 4	SAD day 4
1	soft tissue	1	44	0.44	0.42	0.01	0.33	yes
2	abdominal	18	38	0.58	0.39	0.47	0.04	yes
3	abdominal	2	35	0.17	0.13	0.21	*	yes
4	urogenital	2	35	0.96	0.97	^§^	^§^	^§^
5	abdominal	4	30	0.05	0.25	0.14	0.04	no
6	abdominal	1	37	0.25	0.23	0.17	0.28	yes
7	abdominal	3	35	0.30	0.31	0.12	*	no
8	abdominal	1	24	0.81	*	0.48	*	yes
9	abdominal	1	24	0.26	0.23	0.20	-0.36	no
10	abdominal	38	38	0.25	0.13	0.07	0.07	no
11	abdominal	1	34	0.22	0.42	0.37	*	no
12	abdominal	9	29	0.23	0.13	0.11	0.01	yes
13	abdominal	7	33	0.19	0.25	0.21	0.08	yes
14	abdominal	1	27	0.69	0.39	0.39	0.80	no
15	soft tissue	3	34	0.11	0.19	0.51	0.01	yes
16	abdominal	19	32	0.43	0.49	0.59	0.39	yes
17	abdominal	2	36	0.46	0.27	0.29	0.51	yes
18	abdominal	13	33	0.31	0.20	0.36	0.40	yes
19	abdominal	1	35	0.17	0.43	0.15	0.15	yes
20	soft tissue	2	32	0.64	0.45	-0.53	0.01	yes
21	abdominal	8	29	0.39	0.42	0.88	0.84	yes
22	abdominal	18	31	0.48	0.61	0.30	0.69	yes
23	abdominal	13	28	0.89	0.51	0.28	*	yes
24	abdominal	12	20	0.86	0.84	-0.15	0.05	yes
25	urogenital	4	21	0.07	0.07	0.32	0.04	no
26	abdominal	1	29	0.44	0.46	0.39	0.32	yes
27	abdominal	1	23	0.85	0.73	0.73	0.64	yes
28	abdominal	12	38	0.42	0.62	0.12	0.36	yes
29	abdominal	2	35	0.66	0.15	0.07	0.24	yes
30	abdominal	8	31	0.18	0.32	0.20	0.35	yes

Hospital stay, day of hospital stay at the day of septic onset (days); APACHE II, Acute Physiology and Chronic Health Evaluation score at the first day; Mx, index of cerebrovascular autoregulation; SAD, sepsis-associated delirium measured with confusion-assessment method for the intensive care unit (CAM-ICU). ^§^patient died; *TCD measurement not possible.

**Table 2:** Physiological data

	Day 1	Day 2	Day 3	Day 4
MAP, mmHg	75 ± 9	77 ± 11	80 ± 7	83 ± 10

Mx	0.42 (0.05, 0.96)	0.39 (0.12, 0.97)	0.22 (-0.53, 0.88)	0.28 (-0.36, 0.84)

CI, l/min/m^2^	3.7 (2.3, 7.5)	3.8 (2.6, 5.3)	3.8 (2.3, 7.3)	4.1 (2.7, 7.7)

HR, min^-1^	100 ± 20	92 ± 17	87 ± 15	97 ± 19

Norepinephrine, µg/kg/min	0.34 (0.06, 1.64) n = 30	0.29 (0.03, 1.04) n = 30	0.19 (0.04, 0.73) n = 27	0.17 (0.02, 0.67) n = 18

Dobutamine, µg/kg/min	2,98 (0.69, 6.55) n = 15	2.91 (0.16, 8.20) n = 19	2.05 (0.33, 8.30) n = 19	3.13 (0.52, 5.13) n = 15

Epinephrine, µg/kg/min	0.11 (0.04, 3.90) n = 6	0.15 (0.13, 0.18) n = 2	n = 0	n = 0

Propofol, mg/kg/h	2.1 ± 0.9	1.8 ± 0.7	1.6 ± 0.7	1.4 ± 0.8

Sufentanil, µg/kg/min	0.3 ± 0.1	0.2 ± 0.1	0.2 ± 0.1	0.2 ± 0.1

Core temperature, °C	37.2 ± 1.1	37.2 ± 1.0	37.2 ± 1.0	37.4 ± 0.9

CBFV, cm/s	52 (19, 94)	52 (13, 125)	60 (20, 108)	53 (14, 104)

paO_2_, kPa	15.2 ± 3.9	14.2 ± 3.6	15.3 ± 3.6	14.0 ± 2.8

paCO_2_, kPa	5.7 ± 1.2	5.9 ± 0.9	6.0 ± 1.1	6.4 ± 1.2

CRP, mg/l	209 (44, 491)	308 (31, 478)	214 (26, 397)	146 (16, 329)

PCT, ng/ml	23.5 (1.1, 409.0)	14.0 (2.0, 390.0)	8.5 (1.2, 44.0)	6.2 (0.8, 42.0)

NSE, ng/ml	15.8 (7.1, 40.3)	15.1 (7.0, 65.3)	12.6 (6.5, 28.0)	10.7 (3.2, 47.5)

S100, ng/ml	0.23 (0.11, 3.79)	0.21 (0.07, 0.59)	0.17 (0.07, 0.66)	0.15 (0.05, 0.49)

SOFA, score	12 ± 3	12 ± 3	11 ± 3	10 ± 4

Values are given as mean ± standard deviation or median (minimum, maximum) for distorted data. MAP, mean arterial blood pressure; Mx, autoregulation index; CI, cardiac index; HR, heart rate; n, number of counts of patients needing norepinephrine, dobutamine, and epinephrine; CBFV, cerebral blood flow velocity; paO_2_, arterial partial pressure of oxygen; paCO_2_, arterial partial pressure of carbon dioxide; CRP, C-reactive protein; PCT, procalcitonin; NSE, neuron-specific enolase; SOFA, Sequential Organ Failure Assessment.

### Cerebrovascular autoregulation (AR)

**Figure 1:**
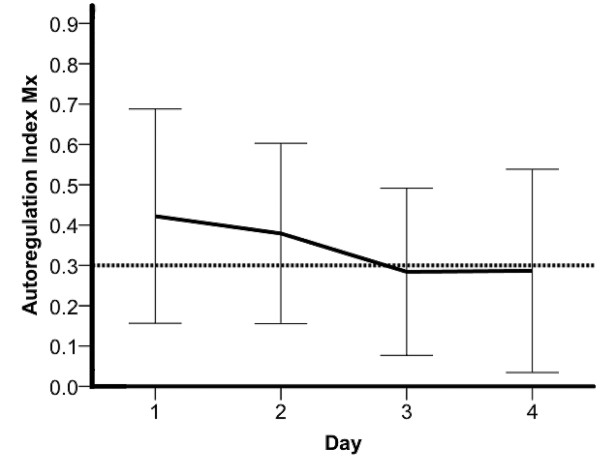
**Mean cerebrovascular autoregulation index (Mx) of all patients during the time of the investigation**. Values above the horizontal line at 0.3 indicate impaired cerebrovascular autoregulation. The lines show mean and first SD.

Of the 30 patients, 25 (83%) showed an impaired AR with an Mx > 0.3 during the first four days, 18/30 (60 %) at day 1, 17/29 (59%) at day 2, 12/29 (41%) at day 3 and 11/24 (46%) at day 4. The highest level of Mx was on the first day with a median of 0.42 (0.05, 0.96) followed by a decrease with a median of 0.30 (-0.53, 0.97) between day 2 and day 3 (Figure 1, Table 2).

### Sepsis-associated delirium and association with AR

**Figure 2:**
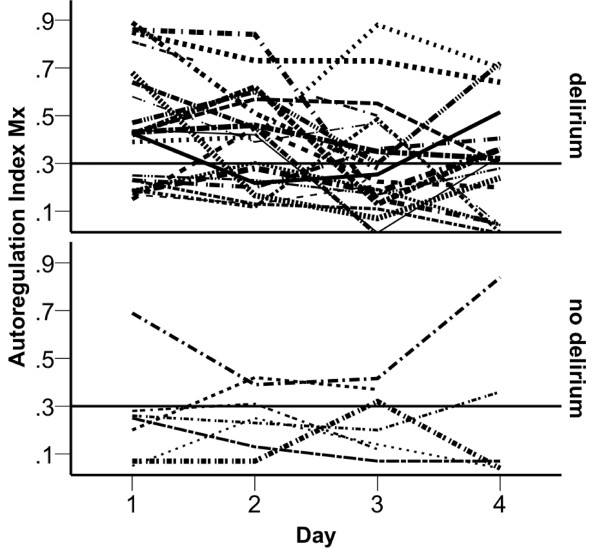
**Cerebrovascular autoregulation index (Mx) during the time of investigation**. Mx values for patients with (upper half) and without (lower half) sepsis-associated delirium (SAD) diagnosed using the confusion assessment method for the ICU (CAM-ICU). Values above the horizontal line at 0.3 indicate impaired cerebrovascular autoregulation.

Diagnosis of SAD using the clinical CAM-ICU at day 4 was performed in 23/29 (76%) patients. EEG could be performed in 18/30 (60%) patients. Of these, 17 patients showed an EEG pattern which is typical for delirium (two with I°, twelve with II°, one with III°, and two patients with IV°). There was no association between the clinical diagnosis of delirium based on CAM-ICU and the EEG (*P *= 0.799). The status of AR at day 1 and the incidence of SAD using the CAM-ICU at day 4 showed an association (*P *= 0.035) (Figure 2), while there was no association between AR and the SAD detected with EEG. Furthermore, there was a positive correlation between age and the occurrence of SAD, while no correlations with APACHE II scores, or maximal serum levels of CRP, PCT, NSE or S100 (Table 3) were found.

**Table 3:** Correlations with sepsis-associated delirium (SAD)

	Impaired AR, n	Age, years	APACHE II score	CRP, mg/l	PCT, ng/ml	NSE, ng/ml	S100, ng/ml
No SAD	1	51 ± 26	30 ± 6	271 ± 129	16 ± 13	15 ± 5	0.5 ± 0.2
SAD	14	64 ± 17	32 ± 6	316 ± 112	49 ± 86	22 ± 13	0.5 ± 0.8
*P*	0.022*	0.023*	0.142	0.373	0.332	0.185	0.886

Mean ± SD, and significance level (*P*) in patients without sepsis-associated delirium (SAD) versus patients with SAD. Impaired AR (cerebrovascular autoregulation), number (n) of patients with impaired AR at day 1; APACHE II, Acute Physiology and Chronic Health Evaluation II; CRP, maximum level of C-reactive protein; PCT, maximum level of procalcitonin; NSE, maximum level of neuron-specific enolase; S100, maximum level of S100. *Significant result at *P *< 0.05, one-way analysis of variance.

### Serum markers

Markers of inflammation correlated at day 2 with the Mx. The maximal serum concentration of S100 during the investigation correlated with the incidence of SAD (-0.401, *P *= 0.028). The serum level of NSE, the severity of illness assessed by the APACHE II and paCO_2 _did not correlate with Mx or SAD (Table 4).

**Table 4:** Correlations with the index of cerebrovascular autoregulation **(**Mx)

	CRP	PCT	NSE	S100	paCO_2_
Mx day 1	0.071	0.285	0.082	-0.066	0.225
Mx day 2	0.525*	0.448*	0.262	0-.058	0.324
Mx day 3	-0.009	-0.176	0.064	-0.054	0.198
Mx day 4	0.302	0.246	-0.083	0.030	0.382

Values are correlation coefficients for association between the Mx and markers of inflammation (CRP, PCT), neuronal damage (NSE), glial damage (S100) and paCO_2_. CRP, C-reactive protein; PCT, procalcitonin; NSE, neuron-specific enolase; paCO_2_, arterial carbon dioxide partial pressure. *P < 0.05, Spearman correlation.

## Discussion

The present study revealed the following main findings: AR was impaired in most of the patients at day 1 followed by recovery during the following days. SAD (CAM-ICU) was detected in 22/29 patients at day 4, which was associated with an impaired AR at day 1. Further minor findings were: the index of AR Mx correlated weakly at day 2 with inflammatory parameters but not with markers of brain damage (NSE, S100). Nevertheless, the marker S100 was elevated in the patients with SAD. There was no correlation between the diagnosis of delirium using CAM-ICU compared to the EEG. Results from the present study therefore, suggest that impairment of AR and SAD is common in ICU patients, and impaired AR might be causative to trigger SAD.

### Cerebrovascular autoregulation

In the present study the TCD-derived index of AR Mx was used for the assessment of the status of AR; this index has been already calculated for many different clinical conditions: in traumatic brain injury, in carotid artery disease, in vasospasm, in volunteers, and in sepsis [10,17]. Mx was furthermore compared to many other accepted tests for assessment of AR: static rate of AR, leg-cuff release, transient hyperaemic response test, autoregulation index, and phase-shift between slow blood pressure and CBFV fluctuations [18]. Measurement of the index Mx need no further manipulation with potential side effects, and therefore, was used in the present study. The index of AR Mx was measured on a daily basis during the first 4 days after the onset of sepsis, showing an increased index Mx and, thereby, a disturbed AR during the first 2 days of sepsis followed by a recovery of AR during the next two days. This time course with initial impaired AR followed by a recovery was also observed in patients with intracranial lesions [19]. The question of whether the disturbance of AR is a clinical sign of critically ill patients in general, or a typical problem of septic patients, cannot be answered by the present study. Nevertheless, the finding that AR is disturbed in septic patients is of clinical relevance, because in patients with impaired AR, the cerebral perfusion pressure has to be closely controlled to avoid cerebral ischaemia due to low cerebral perfusion pressures or haemorrhage, and brain oedema with high cerebral perfusion pressures. Therefore, the findings of the present study suggest a tightly controlled cerebral perfusion pressure in septic patients during the first 2 days.

### Sepsis-associated delirium

SAD occurs in many septic patients and may be a risk factor for morbidity [2]. This is in line with the results of the present study where 77 % of all patients developed SAD. To assess SAD, several methods can be used. The CAM-ICU is the most popular, however, it can only be applied during interruption of the sedative medication and with RASS score > -2 [15]. Assessment of delirium using EEG has been successfully performed in 69 septic patients who were awake [16]. However, in the present study the results for the diagnosis of SAD using the CAM-ICU and the EEG did not correlate. Possibly the individual sedation during the recording of EEG was not comparable between the two studies, as in the present study patients were sedated, while in the former study the patients were awake. Propofol can dose-dependently affect the EEG directly by an increase of slow waves. However, it is also possible that the EEG changes during sepsis do not reflect the clinical signs of SAD. This suggests that the validity of EEG to detect the occurrence of SAD in slightly sedated patients should be prospectively evaluated in a larger series of patients using a more standardised sedation. In conclusion, the diagnosis of SAD is defined by mental changes and a clinical method to detect these psychiatric changes is therefore, recommended for ICU patients, especially the CAM-ICU [15].

### Association of AR and SAD

Disturbed AR may lead to global brain ischaemia due to hypotension in the absence of adequate regulatory compensation. This ischaemia may be a trigger for brain dysfunction like SAD. In the present study an association between impaired AR at day 1 and SAD at day 4 was shown. An investigation including 10 patients with pathological EEG results and sepsis showed intact CO_2 _reactivity [20] and therefore, assumed that no correlation between SAD and cerebrovascular haemodynamic exists. However, the CO_2_-dependent autoregulation of CBF is much more resistant towards influences compared to the blood pressure-dependent CBF autoregulation, which was tested in the present study. This might explain why the CO_2 _reactivity study showed no impaired vasomotor reactivity, while in the present study the MAP-dependent AR was impaired in patients with SAD. On the other hand, in 16 septic patients the index Mx was measured once during the first 48 hours after stabilisation and disturbance of AR was correlated with the diagnosis of SAD (CAM-ICU) [10]. In another study the cerebrovascular reactivity to acetazolamide was impaired in 14 patients with sepsis and disturbance of consciousness compared with a control group [21]. This association between AR and SAD was confirmed in the present study, investigating 30 septic patients over a period of 4 days. Patients with severe sepsis and septic shock showed an association between impaired AR and SAD. Therefore, impaired AR might be an influencing factor for the development of SAD in these patients. These results suggest that fluctuations in blood pressure, which regularly occur during the first days of sepsis, in combination with the impaired AR might result in global brain hypo- or hyperperfusion and cause SAD. This assumption cannot be verified using the presented data but the detected association between impaired AR and SAD further supports the need for trials investigating whether a tight control of arterial blood pressure, avoiding hypo- and hypertension during the first days of sepsis, could reduce the incidence of SAD.

### Serum markers

Levels of CRP and PCT, validated markers of inflammation, correlated only at day 2 with the index of AR Mx. Possibly, disturbance of AR is fostered by the underlying inflammation caused by endothelial dysfunction due to endotoxins from bacteria, or cytokines like ICAM-1 from the immune response [22,23]. The peak of CRP and PCT after onset of infection may be delayed and represents the severity of inflammation mainly at day 2. This would explain why the correlation of Mx with CRP and PCT could be found only at day 2.

### Limitations of the study

The sedation management with propofol in combination with sufentanil did not influence AR in patients under general anaesthesia and after head injury [24-26]. It is therefore, unlikely that the sedative regime affected AR during this investigation.

To minimize any bias the same technical equipment was always used. TCD is an operator-dependent tool because of the dependency of the angle of insonation. However, for the assessment of AR only the relation of changes in CBFV in correspondence to changes in MAP was used rather than the absolute values of CBFV.

The levels of paCO_2 _in the present study were in the upper normal range and increased from 43 ± 9 mmHg at day 1 to 48 ± 9 mmHg at day 4, and might have influenced the AR [27]. This was tolerated because of the benefit of spontaneous breathing for the patient. However, the influence of this increase in paCO_2 _on the present results should be acceptable as high paCO_2 _further impairs AR. Therefore, the observed recovery of AR until day 4 should be even more pronounced if paCO_2 _was held constant during the investigation.

Another possible influencing factor is hyperthermia, because an increase of core temperature above 40°C leads to impairment of AR [28]. However, in the present study the temperature was between 34.2 and 39.4°C (mean 37.2 °C) throughout the measurement and therefore, should not be a relevant influencing factor.

## Conclusions

The present study showed that AR is impaired in the majority of patients with severe sepsis and septic shock during the first 2 days. Furthermore, SAD was frequently diagnosed in these patients (76% of all investigated patients). Impairment of AR was associated with the occurrence of SAD, suggesting that AR is one of the factors contributing to SAD. This should be taken into account for the treatment of these patients.

## Key messages

• Cerebrovascular AR is impaired in 83% of patients with severe sepsis and septic shock

• Among patients with severe sepsis and septic shock, 76% have sepsis-associated delirium

• Impaired cerebrovascular AR possibly triggers sepsis-associated delirium

• Possibly, mean arterial blood pressure should be tightly controlled during the first 2 days in septic patients

## Abbreviations

ANOVA: analysis of variance; APACHE: Acute Physiology and Chronic Health Evaluation; AR: cerebrovascular autoregulation; CAM-ICU: confusion assessment method for the intensive care unit; CBF: cerebral blood flow; CBFV: cerebral blood flow velocity; CI: cardiac index; CRP: C-reactive protein; EEG: electroencephalography; HR: heard rate; ICU; intensive care unit; MAP: mean arterial pressure; Mx: Index of cerebrovascular autoregulation; NSE: neuron specific enolase; paCO_2_: arterial partial pressure of carbon dioxide; paO_2_: arterial partial pressure of oxygen; PCT: procalcitonin; PiCCO: pulse contour cardiac output; RASS: Richmond Agitation and Sedation Scale; SAD: sepsis-associated delirium; SOFA: Sequential Organ Failure Assessment; TCD: transcranial Doppler.

## Competing interests

The authors declare that they have no competing interests.

## Authors' contributions

PS, KUK, LF, DC performed the TCD and CAM-ICU, PS and KJW performed the EEG and KJW interpreted the EEG; PS, DC, KE drafted the document; PS, MD, CW and KE participated in planning the design of the study; MB, LF and PS performed statistical analysis. All authors read and approved the final manuscript.

## Authors' information

KJW is a board-certified neurologist with special experience in EEG; MB is statistician with experience in clinical trials; LF is medical student and intensive care nurse; PS is an anaesthesiologist and intensive care physician with experience in neurology and neurophysiologic monitoring; KUK, DC, KE, MD and CW are anaesthesiologists with experience in intensive care and neurophysiologic monitoring.

## References

[B1] Shehabi Y Riker RR Bokesch PM Wisemandle W Shintani A Ely EW Delirium duration and mortality in lightly sedated, mechanically ventilated intensive care patients Crit Care Med 2010 16 2311 2318 10.1097/CCM.0b013e3181f85759 20838332

[B2] Ebersoldt M Sharshar T Annane D Sepsis-associated delirium Intensive Care Medicine 2007 16 941 950 10.1007/s00134-007-0622-2 17410344

[B3] Cerejeira J Firmino H Vaz-Serra A Mukaetova-Ladinska EB The neuroinflammatory hypothesis of delirium Acta Neuropathol 2010 16 737 754 10.1007/s00401-010-0674-1 20309566

[B4] Iacobone E Bailly-Salin J Polito A Friedman D Stevens RD Sharshar T Sepsis-associated encephalopathy and its differential diagnosis Crit Care Med 2009 16 S331 336 20046118 10.1097/CCM.0b013e3181b6ed58

[B5] Sharshar T Carlier R Bernard F Guidoux C Brouland JP Nardi O de la Grandmaison GL Aboab J Gray F Menon D Annane D Brain lesions in septic shock: a magnetic resonance imaging study Intensive Care Med 2007 16 798 806 10.1007/s00134-007-0598-y 17377766

[B6] Taccone FS Su F Pierrakos C He X James S Dewitte O Vincent J-L De Backer D Cerebral microcirculation is impaired during sepsis: an experimental study Crit Care 2010 16 R140 10.1186/cc9205 20667108 PMC2945121

[B7] Semmler A Hermann S Mormann F Weberpals M Paxian SA Okulla T Schafers M Kummer MP Klockgether T Heneka MT Sepsis causes neuroinflammation and concomitant decrease of cerebral metabolism J Neuroinflammation 2008 16 38 10.1186/1742-2094-5-38 18793399 PMC2553764

[B8] Terborg C Schummer W Albrecht M Reinhart K Weiller C Rother J Dysfunction of vasomotor reactivity in severe sepsis and septic shock Intensive Care Med 2001 16 1231 1234 10.1007/s001340101005 11534574

[B9] Sharshar T Annane D de la Grandmaison GL Brouland JP Hopkinson NS Francoise G The neuropathology of septic shock Brain Pathol 2004 16 21 33 14997934 10.1111/j.1750-3639.2004.tb00494.x PMC8095740

[B10] Pfister D Siegemund M Dell-Kuster S Smielewski P Rüegg S Strebel SP Marsch SC Pargger H Steiner LA Cerebral perfusion in sepsis-associated delirium Crit Care 2008 16 R63 10.1186/cc6891 18457586 PMC2481444

[B11] Levy MM Fink MP Marshall JC Abraham E Angus D Cook D Cohen J Opal SM Vincent JL Ramsay G 2001 SCCM/ESICM/ACCP/ATS/SIS International Sepsis Definitions Conference Intensive Care Med 2003 16 530 538 12664219 10.1007/s00134-003-1662-x

[B12] Dellinger RP Levy MM Carlet JM Bion J Parker MM Jaeschke R Reinhart K Angus DC Brun-Buisson C Beale R Calandra T Dhainaut JF Gerlach H Harvey M Marini JJ Marshall J Ranieri M Ramsay G Sevransky J Thompson BT Townsend S Vender JS Zimmerman JL Vincent JL Surviving Sepsis Campaign: international guidelines for management of severe sepsis and septic shock: 2008 Intensive Care Med 2008 16 17 60 10.1007/s00134-007-0934-2 18058085 PMC2249616

[B13] Czosnyka M Smielewski P Kirkpatrick P Menon DK Pickard JD Monitoring of cerebral autoregulation in head-injured patients Stroke 1996 16 1829 1834 10.1161/01.STR.27.10.1829 8841340

[B14] Sorrentino E Budohoski KP Kasprowicz M Smielewski P Matta B Pickard JD Czosnyka M Critical thresholds for transcranial Doppler indices of cerebral autoregulation in traumatic brain injury Neurocrit Care 2011 16 188 193 10.1007/s12028-010-9492-5 21181299

[B15] Plaschke K von Haken R Scholz M Engelhardt R Brobeil A Martin E Weigand MA Comparison of the confusion assessment method for the intensive care unit (CAM-ICU) with the Intensive Care Delirium Screening Checklist (ICDSC) for delirium in critical care patients gives high agreement rate(s) Intensive Care Med 2008 16 431 436 10.1007/s00134-007-0920-8 17994221

[B16] Young GB Bolton CF Archibald YM Austin TW Wells GA The electroencephalogram in sepsis-associated encephalopathy J Clin Neurophysiol 1992 16 145 152 10.1097/00004691-199201000-00016 1552002

[B17] Steiner LA Pfister D Strebel SP Radolovich D Smielewski P Czosnyka M Near-Infrared Spectroscopy can Monitor Dynamic Cerebral Autoregulation in Adults Neurocrit Care 2009 16 122 128 10.1007/s12028-008-9140-5 18807218

[B18] Czosnyka M Brady K Reinhard M Smielewski P Steiner LA Monitoring of Cerebrovascular Autoregulation: Facts, Myths, and Missing Links Neurocrit Care 2009 16 373 386 10.1007/s12028-008-9175-7 19127448

[B19] Schramm P Klein KU Pape M Berres M Werner C Kochs E Engelhard K Serial measurement of static and dynamic cerebrovascular autoregulation after brain injury J Neurosurg Anesthesiol 2011 16 41 44 10.1097/ANA.0b013e3181f35854 21252706

[B20] Thees C Kaiser M Scholz M Semmler A Heneka MT Baumgarten G Hoeft A Putensen C Cerebral haemodynamics and carbon dioxide reactivity during sepsis syndrome Crit Care 2007 16 R123 10.1186/cc6185 18045492 PMC2246217

[B21] Szatmari S Vegh T Csomos A Hallay J Takacs I Molnar C Fulesdi B Impaired cerebrovascular reactivity in sepsis-associated encephalopathy studied by acetazolamide test Crit Care 2010 16 R50 10.1186/cc8939 20356365 PMC2887164

[B22] Hofer S Bopp C Hoerner C Plaschke K Faden RM Martin E Bardenheuer HJ Weigand MA Injury of the blood brain barrier and up-regulation of icam-1 in polymicrobial sepsis J Surg Res 2008 16 276 281 10.1016/j.jss.2007.07.021 18164036

[B23] Papadopoulos MC Davies DC Moss RF Tighe D Bennett ED Pathophysiology of septic encephalopathy: a review Crit Care Med 2000 16 3019 3024 10.1097/00003246-200008000-00057 10966289

[B24] Engelhard K Werner C Möllenberg O Kochs E S(+)-ketamine/propofol maintain dynamic cerebrovascular autoregulation in humans Can J Anaesth 2001 16 1034 1039 10.1007/BF03016597 11698326

[B25] Engelhard K Werner C Mollenberg O Kochs E Effects of remifentanil/propofol in comparison with isoflurane on dynamic cerebrovascular autoregulation in humans Acta Anaesthesiol Scand 2001 16 971 976 10.1034/j.1399-6576.2001.450809.x 11576048

[B26] Ogawa Y Iwasaki K-I Aoki K Gokan D Hirose N Kato J Ogawa S The different effects of midazolam and propofol sedation on dynamic cerebral autoregulation Anesth Analg 2010 16 1279 1284 10.1213/ANE.0b013e3181f42fc0 20881283

[B27] Piechnik SK Yang X Czosnyka M Smielewski P Fletcher SH Jones AL Pickard JD The continuous assessment of cerebrovascular reactivity: a validation of the method in healthy volunteers Anesth Analg 1999 16 944 949 10512269 10.1097/00000539-199910000-00023

[B28] Cremer OL Diephuis JC van Soest H Vaessen PH Bruens MG Hennis PJ Kalkman CJ Cerebral oxygen extraction and autoregulation during extracorporeal whole body hyperthermia in humans Anesthesiology 2004 16 1101 1107 10.1097/00000542-200405000-00011 15114206

